# Supplementary material for: Tuning the interplay between nematicity and spin fluctuations in Na1−xLixFeAs superconductors
Source: Nat Commun. 2018 May 30;9:2139. doi: 10.1038/s41467-018-04471-7 (PMC5976654; doi:10.1038/s41467-018-04471-7)
Supplement: Supplementary file 1 — Supplementary Information [file 41467_2018_4471_MOESM1_ESM.pdf]

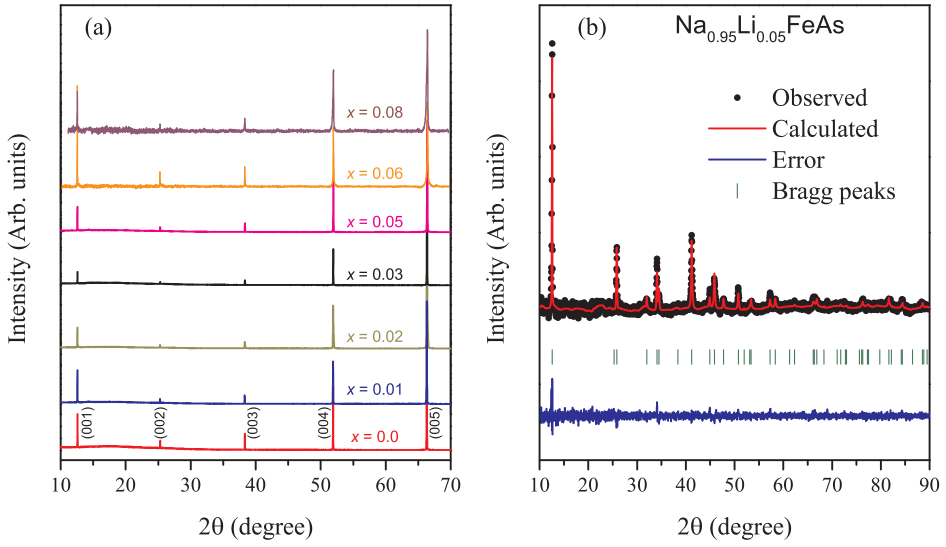

Supplementary Figure 1. X-ray diffraction data. (a) The single crystalline x-ray diffraction data of selected $Na_{1-x}Li_xFeAs$ single crystals. $(00l)$ reflection peaks can only be seen in the XRD patterns suggesting the absence of any other impurity phase. The diffraction patterns could be successfully refined by the tetragonal $P4/nmm$ structure as in the parent NaFeAs (ref. 1) (b) Powder diffraction pattern of a ground $Na_{0.95}Li_{0.05}FeAs$ single crystals.

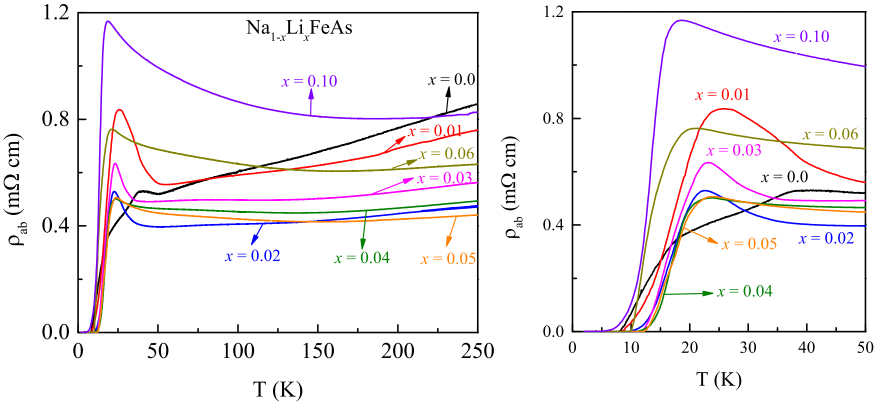

Supplementary Figure 2. Original resistivity data. Temperature dependence of the in-plane resistivity in $Na_{1-x}Li_xFeAs$ single crystals for different Li concentration x.

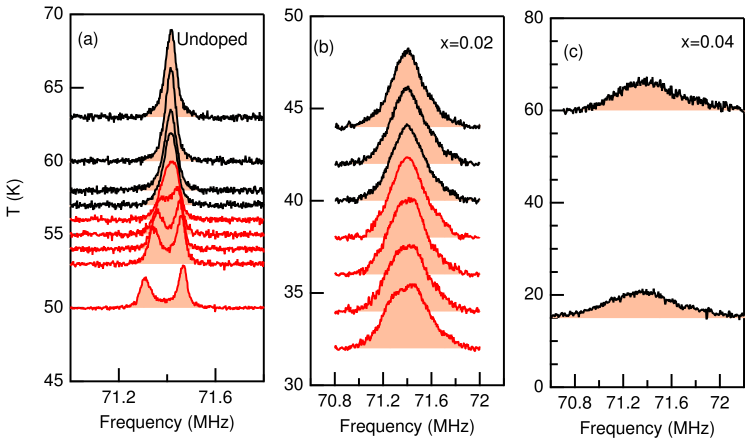

Supplementary Figure 3. Determination of the nematic transition temperature by NMR. Temperature dependence of the ^{75}As satellite line for (a) $x = 0$, (b) 0.02, and (c) 0.04. The line splitting caused by nematic order was observed for $x = 0$ and 0.02, which allows us to determine the nematic transition temperature. For $x = 0.04$, the splitting is not detected, probably due to the larger broadening of the line than the splitting.

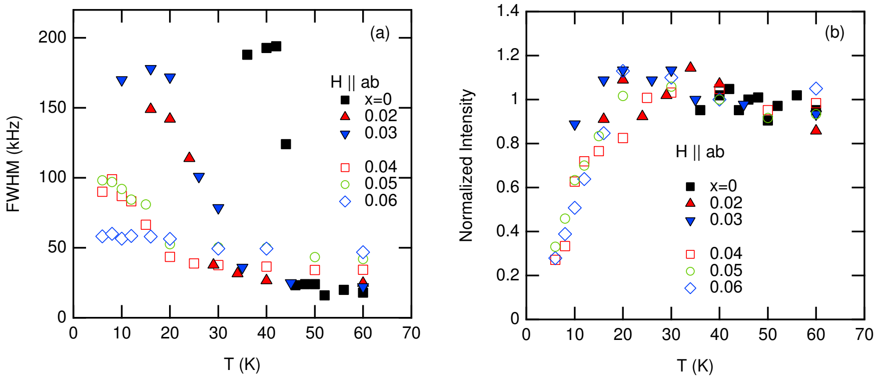

Supplementary Figure 4. Detailed analysis of ^{75}As spectra. (a) The full width at half maximum (FWHM) as a function of doping and temperature. For $x \geq 0.04$, the FWHM is weakly broadened down to low temperatures. Note that for $x = 0.06$ there is no broadening of the line at all, while the sharp peak of $(T_1T)^{-1}$ is clearly observed. For $x = 0$, the FWHM data were multiplied by 4 to compare directly with those of doped samples. (b) Normalized signal intensity as a function of doping and temperature. While the signal intensity does not change within experimental error for $x < 0.03$, it is notably suppressed below T_0 for $x \geq 0.04$.

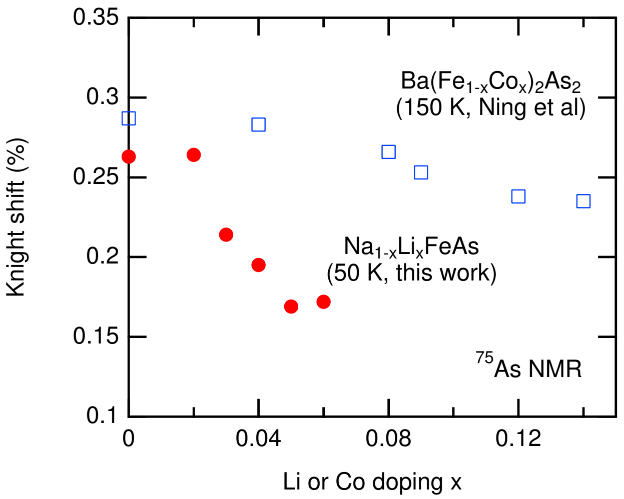

Supplementary Figure 5. Sharp reduction of the Knight shift near $x = 0.03$. Doping dependence of the Knight shift $\mathcal{K}$ at 50 K is compared with data in Ba(Fe$_{1-x}$Co$_x$)$_2$As$_2$ at 150 K (taken from ref. 2).

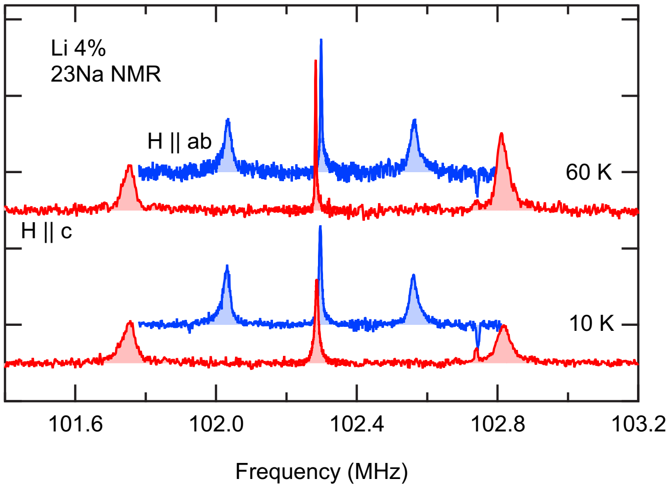

Supplementary Figure 6. Comparison of ^{23}Na spectra above and below T_0 for $x = 0.04$. The spectrum does not reveal a notable difference in the ordered phase, unlike the ^{75}As results, reflecting that the ^{75}As effectively probes the Fe sites due to the much stronger hyperfine coupling.

[1] D. R. Parker, M. J. Pitcher, P. J. Baker, I. Franke, T. Lancaster, S. J. Blundell, and S. J. Clarke, "Structure, antiferromagnetism and superconductivity of the layered iron arsenide NaFeAs," Chem. Commun. , 2189–2191 (2009).

[2] F. L. Ning, K. Ahilan, T. Imai, A. S. Sefat, M. A. McGuire, B. C. Sales, D. Mandrus, P. Cheng, B. Shen, and H.-H Wen, "Contrasting Spin Dynamics between Underdoped and Overdoped $Ba(Fe_{1-x}Co_x)_2As_2$," Phys. Rev. Lett. **104**, 037001 (2010).
